# The Amino-Terminus of Nitric Oxide Sensitive Guanylyl Cyclase α_1_ Does Not Affect Dimerization but Influences Subcellular Localization

**DOI:** 10.1371/journal.pone.0025772

**Published:** 2011-09-30

**Authors:** Jan R. Kraehling, Mareike Busker, Tobias Haase, Nadine Haase, Markus Koglin, Monika Linnenbaum, Soenke Behrends

**Affiliations:** 1 Department of Pharmacology, Toxicology and Clinical Pharmacy, University of Brunswick - Institute of Technology, Brunswick, Germany; 2 Berlin-Brandenburg Center for Regenerative Therapies (BCRT), Charité Universitätsmedizin Berlin, Campus Virchow-Klinikum, Berlin, Germany; 3 Experimental and Clinical Research Center and Max-Delbrück Center for Molecular Medicine, Berlin, Germany; 4 HEPTARES Therapeutics, Hertfordshire, United Kingdom; Griffith University, Australia

## Abstract

**Background:**

Nitric oxide sensitive guanylyl cyclase (NOsGC) is a heterodimeric enzyme formed by an α- and a β_1_-subunit. A splice variant (C-α_1_) of the α_1_-subunit, lacking at least the first 236 amino acids has been described by *Sharina* et al. 2008 and has been shown to be expressed in differentiating human embryonic cells. *Wagner* et al. 2005 have shown that the amino acids 61–128 of the α_1_-subunit are mandatory for quantitative heterodimerization implying that the C-α_1_-splice variant should lose its capacity to dimerize quantitatively.

**Methodology/Principal Findings:**

In the current study we demonstrate preserved quantitative dimerization of the C-α_1_-splice by co-purification with the β_1_-subunit. In addition we used fluorescence resonance energy transfer (FRET) based on fluorescence lifetime imaging (FLIM) using fusion proteins of the β_1_-subunit and the α_1_-subunit or the C-α_1_ variant with ECFP or EYFP. Analysis of the respective combinations in HEK-293 cells showed that the fluorescence lifetime was significantly shorter (≈0.3 ns) for α_1_/β_1_ and C-α_1_/β_1_ than the negative control. In addition we show that lack of the amino-terminus in the α_1_ splice variant directs it to a more oxidized subcellular compartment.

**Conclusions/Significance:**

We conclude that the amino-terminus of the α_1_-subunit is dispensable for dimerization *in-vivo* and *ex-vivo*, but influences the subcellular trafficking.

## Introduction

Nitric oxide sensitive guanylyl cyclase is the physiological receptor for nitric oxide (NO) and nitric oxide releasing drugs. Its second messenger cyclic GMP is crucial for vasodilatation, penile erection, platelet disaggregation and neurotransmission. The heterodimeric enzyme is formed by either an α_1_- or an α_2_-subunit and a β_1_-subunit. Dimerization of the enzyme is a prerequisite for its catalytic activity, because both the α- as well as the β_1_-subunit provide essential residues for the conversion of GTP to cGMP [1]. There is conflicting evidence which parts of the subunits are mandatory for quantitative heterodimerization. Previous studies [2], [3] show inconsistent results with respect to the requirement of the α_1_ subunit amino-terminus which is missing in the naturally occurring C-α_1_ splice variant [4]. Since the occurrence of this splice variant has been linked to differentiation of human embryonic stem cells [5], the capacity of the C-α_1_ splice variant to heterodimerize and thus form a functionally active enzyme, is of biological importance.

Because of the controversial nature of the question, we investigated the dimerization capacity using two independent experimental approaches. First we used a purification method of the NOsGC β_1_-subunit and looked for co-purification of the α_1_ variants. With the second experimental approach we examined fluorescence resonance energy transfer (FRET) based on fluorescence lifetime imaging (FLIM) in intact cells using fusion proteins of the respective subunits with fluorescent proteins. While analyzing the fluorescence lifetimes of the respective NOsGC-subunits, we serendipitously discovered that the C-α_1_ splice isoform, shows a unique subcellular distribution. Using a fluorescent tagged marker for the endoplasmic reticulum (ER), we conclude, that the C-α_1_ isoform is located at the ER. As it is well known, that the redox state of the ER is relatively more oxidized than the cytosol [6], we performed a ratiometric analysis of the redox properties of the C-α_1_ protein by using a redox sensor (Grx1-roGFP2) and identified, that the protein is not only distributed in a different manner than the wild type, but also located in a more oxidized environment. Co-expression of the β_1_-subunit restored the cytosolic localization of the C-α_1_ splice isoform, but led to a nuclear localization that was not found for the canonical α_1_/β_1_ heterodimer. We could show that the C-α_1_ splice isoform retains its ability to heterodimerize quantitatively with the β_1_-subunit ex vivo and in intact cells, despite lack of amino-terminal amino acids that were thought to be important for heterodimerization [2]. In addition, we observed that the fluorescent fusion of the C-α_1_ splice subunit is directed to a more oxidized subcellular compartment, while the respective C-α_1_/β_1_ heterodimer shows a cytosolic and nuclear localization.

## Results

### α_1_ΔN_259_ will be expressed by the C-α_1_ splice form

**Figure 1:**
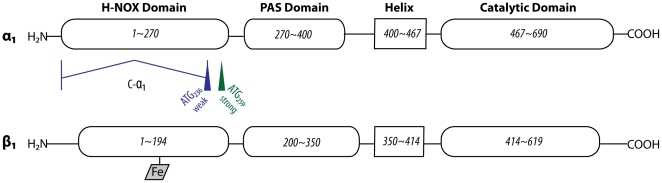
Assumed formation of α_1_ΔN_259_ deletion through C-α_1_ splice variant of guanylyl cyclase. The graphic is a modified version of the domain architecture model by *Derbyshire* and *Marletta* [34].

**Figure 2:**
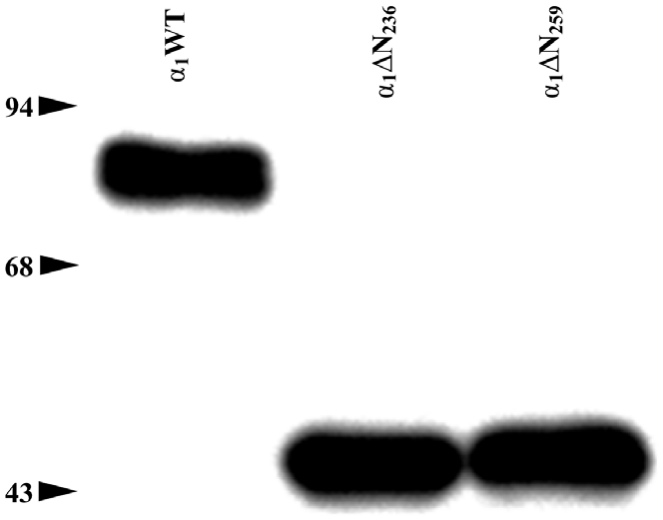
Characterization of expression of the α_1_-subunits by Western-blot analysis in cytosolic fractions of *Sf-*9 cells infected with the respective variants of the α_1_-subunits. All lanes were loaded with 40 µg protein of the cytosolic fractions. The experiments were repeated at least three times and one representative result is shown. [molecular weights predicted from their amino acid sequences: α_1_WT, 77.6 kDa; α_1_ΔN_236_, 51.2 kDa; α_1_ΔN_259_, 48 kDa].

Analyzing the C-α_1_ splice-variant described by *Sharina* et al. [4] and designated α_1_ΔN_240_ shows that the first initiation codon after splicing would either form an α_1_ΔN_236_ or an α_1_ΔN_259_ variant because these represent the only methionines with an open reading frame in the human sequence (Fig. 1 and Data S2). In a recently published review by *Sharina* et al. 2011, the authors have adapted our numbering [7]. In a previous study by *Koglin* and *Behrends* 2003 [3], we investigated intensely the α_1_ΔN_236_ truncation in comparison to the α_1_ΔN_259_ truncation. Because neither enzyme activity, substrate-dependency (GTP), nor dose-effect-curve of nitric oxide differed significantly from α_1_ΔN_259_, it was suggested by the reviewers to remove the redundant data for the α_1_ΔN_236_ truncation. Since there was no difference in molecular weight between the α_1_ΔN_236_ and α_1_ΔN_259_ variant (Fig. 2), we now assume that the recognition of the translation initiation site of ATG_259_ is dominant over ATG_236_ in the human sequence. We thus suggest that the C-α_1_ splice variant leads to the formation of a subunit with an α_1_ΔN_259_ deletion.

### Establishment of a novel one-step-purification protocol

We performed a new one-step-purification of the soluble guanylyl cyclase using the *Strep* Tag II, which results in 1 mg purified NOsGC from 1,000 ml *Sf-*9 culture medium. The yield calculated by using the total activities of cytosol and purified protein is ≈25 %. The purification factor is ≈125-fold and results in a more than 95 % purity of the enzyme determined by Coomassie stained gels.

### Analysis of the heterodimerization of C-α_1_ by co-purification with β_1_-*S*

**Figure 3:**
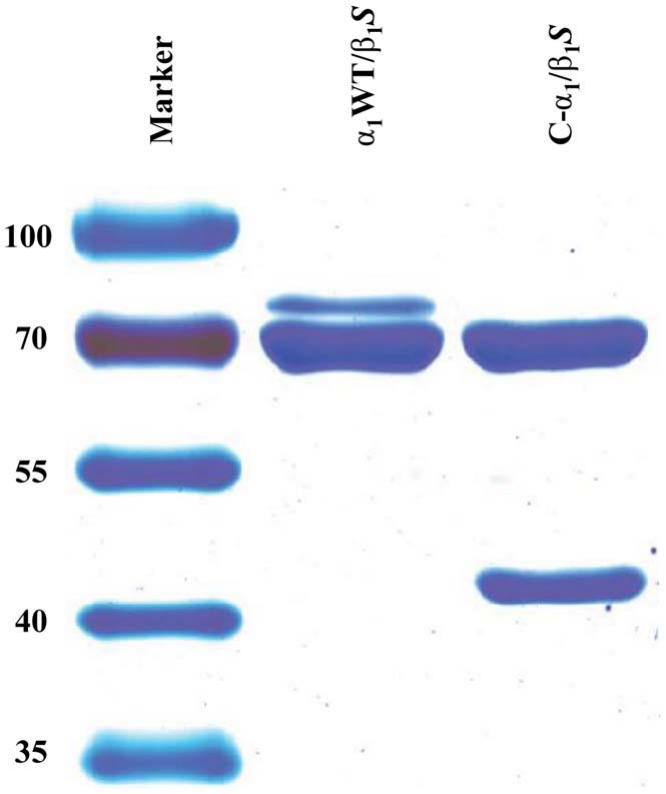
SDS-PAGE analysis of NOsGC variants. 5 µg of each purified enzyme was electrophoretically separated by SDS-Page and stained with Coomassie Blue. The experiments were repeated at least three times and one representative result is shown. [molecular weights predicted from their amino acid sequences: α_1_WT, 77.6 kDa; C-α_1_, 48 kDa; β_1_ *S*, 72.2 kDa].

**Figure 4:**
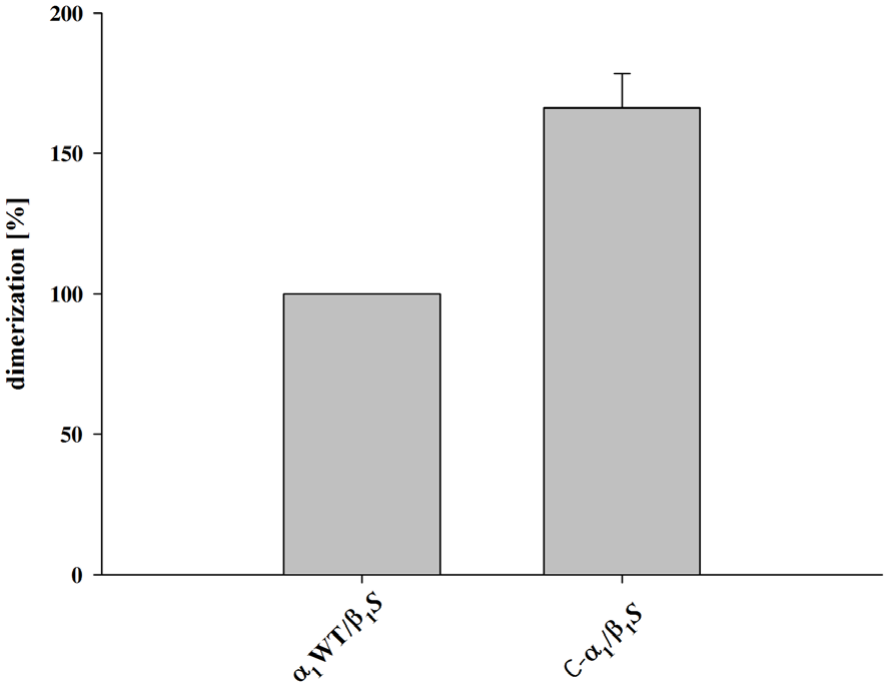
Densitometric analysis of dimerization. For quantification of the dimerization the optical densities of the Coomassie-stained bands were measured. A: The values obtained for α_1_ were normalized to β_1_ in the same lane. Dimerization was calculated in percentage of α_1_WT/β_1_ *S*. Data are expressed as means ± SEM.

**Figure 5:**
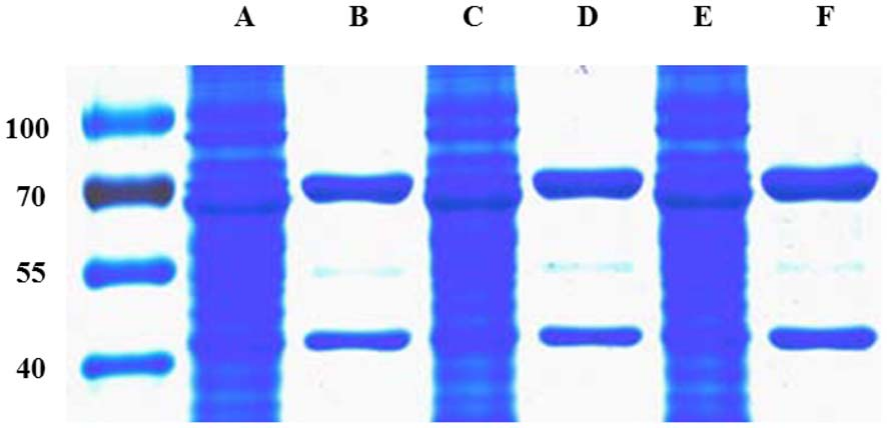
Coomassie staining of cytosolic fractions and purified proteins. 50 µg Cytosol of cytosolic fraction or 5 µg of purified enzyme was electrophoretically separated by SDS-PAGE and stained with Coomassie Blue (a representative gel is shown). Lane A, cytosolic fraction of C-α_1_/β_1_ *S* expressed without heme-supplement and without lipid medium supplement. Lane B, purified C-α_1_/β_1_ *S* expressed without heme-supplement and without lipid medium supplement. Lane C, cytosolic fraction of C-α_1_/β_1_ *S* expressed only with heme-supplement and without lipid medium supplement. Lane D, purified C-α_1_/β_1_ *S* expressed with heme-supplement and without lipid medium supplement. Lane E, cytosolic fraction of C-α_1_/β_1_ *S* expressed only with heme-supplement and with lipid medium supplement. Lane F, purified C-α_1_/β_1_ *S* expressed with heme-supplement and with lipid medium supplement.

Using a carboxy-terminally *Strep* tagged β_1_-subunit of NOsGC, we could show that both α_1_-isoforms dimerize with the β_1_-subunit (Fig. 3). Densitometric analysis and graphical representation as described in [2] shows that C-α_1_ forms heterodimers not less but more effectively with a relative value of 166.2 % (±12.2 %) compared with α_1_WT (Fig. 4). Alternatively the ratio of the band intensities of the α_1_WT variants to the β_1_- bands were 0.75±0.11 for C-α_1_/β_1_ and 0.43±0.06 for α_1_/β_1_. The excess of the β_1_ subunit is due to the fact that purification was performed with a *Strep* Tag II attached to the β_1_-subunit as described in [2]. Because our findings contradict the central message of the paper by *Wagner* at al. 2005 although the experimental approach was very similar [2], we tested more subtle differences. We expressed *Sf-*9 cells without any supplement, only with hemin (4 mg/l) and with both hemin and lipid supplement as in [2], the latter being used to reduce the shear forces during *Sf-*9 cell culture. After expression under these conditions, we purified the enzyme complex and performed a SDS-PAGE and the gel was stained with Coomassie Blue according to the protocol by *Kang* et al. [8]. There was no negative effect of either of the supplements on the heterodimerization of C-α_1_/β_1_ *S* (Fig. 5) confirming our finding that the C-α_1_ splice-variant retains its ability to heterodimerize under different experimental conditions.

**Figure 6:**
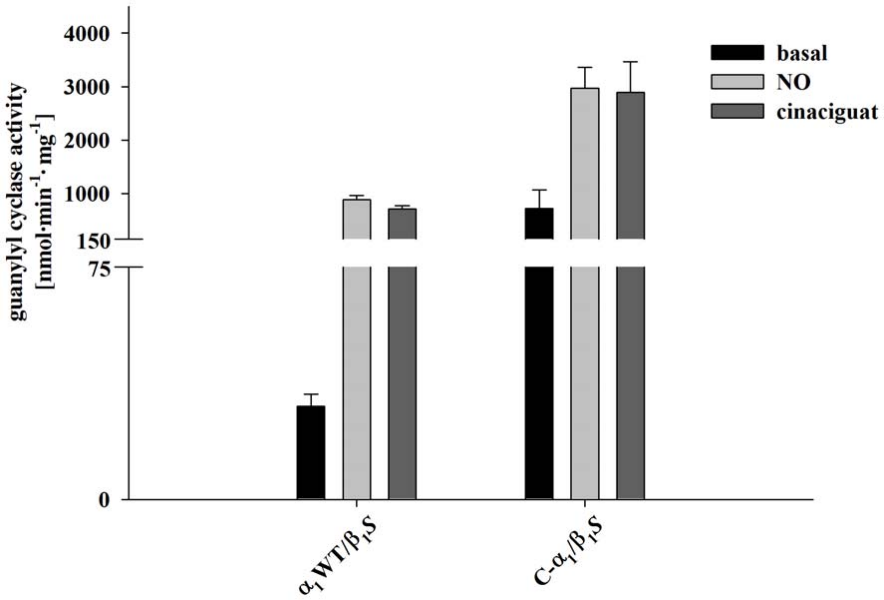
Guanylyl cyclase activity of purified variants of the enzyme. Specific activity was measured under basal conditions (*black column*), in the presence of 100 µM NO (DEA/NO, *white column*) and in the presence of 10 µM cinaciguat (*gray columns*). Data are expressed as means ± SEM. The experiments were repeated at least three times and one representative result is shown.

**Figure 7:**
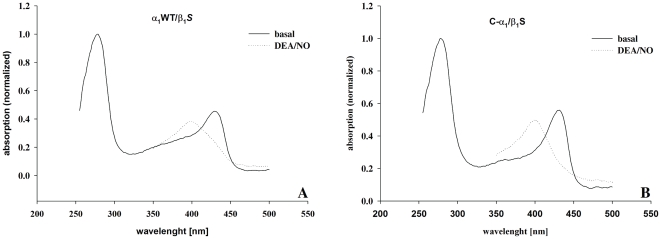
Spectroscopic analysis of purified cGC enzyme complexes. Spectroscopic analysis shows absorption values at basal (*solid line*) or NO-stimulated (100 µM DEA/NO, *dotted line*) conditions. A: α_1_WT/β_1_ *S*. B: C-α_1_/β_1_ *S*.

Guanylyl cyclase activity of the purified C-α_1_/β_1_ and α_1_/β_1_ heterodimers was measured under basal conditions, in the presence of NO (100 µM DEA/NO) or in the presence of 10 µM cinaciguat. Consistent with the formation of more functional heterodimers for C-α_1_/β_1_ versus α_1_/β_1_ in *Sf-*9 cell culture, enzyme activity was higher for the splice variant under all experimental conditions (Fig. 6). Spectroscopic analysis shows that both C-α_1_/β_1_ than α_1_/β_1_ contain a significant amount of heme (Fig. 7) which is consistent with their responsiveness to nitric oxide (see Fig. 6).

### Analysis of the heterodimerization of C-α_1_ by FLIM-FRET

**Figure 8:**
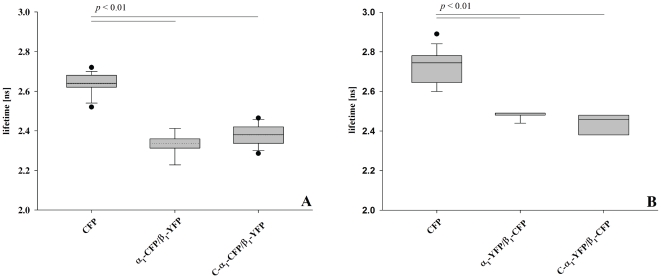
Fluorescence lifetimes of the FRET donor ECFP in HEK-293 cells, expressing donor only, and fluorescent tagged heterodimeric NOsGC variants. Mean fluorescence lifetimes of ECFP were measured in HEK-293 cells at 37°C, 48 h post transfection with the respective recombinant constructs. A: α_1_-variants as fluorescence-donor. B: α_1_-variants as fluorescence-acceptor. Data are presented in box and whiskers plots showing the 25^th^ percentiles, 75^th^ percentile and median as box with the mean value as dotted line. Whiskers represent the 5^th^ and 95^th^ percentile, dots are outliers. Fluorescent tagged NOsGC variants show significantly reduced fluorescent lifetimes due to FRET.

In order to test whether the unexpected formation of more functional heterodimers with the β_1_ subunit for C-α_1_ versus the canonical α_1_ subunit is also seen in intact cells we used fluorescence resonance energy transfer (FRET) based on fluorescence lifetime imaging. The α_1_-subunit and C-α_1_-subunit were fused with ECFP and the β_1_-subunit with EYFP in analogy to a previous FRET study by our group [9]. Analysis of the respective combinations in HEK-293 cells showed that the fluorescence lifetime was significantly shorter for α_1_/β_1_ and C-α_1_/β_1_ than the negative control (Fig. 8A). This indicates that heterodimerization of both α_1_ variants with the β_1_ subunit bring the respective fused fluorescent proteins in a physical distance below 80 Å [10]. As an additional control, FLIM-FRET experiments were performed with constructs where the fluorescent proteins were exchanged (Fig. 8B). Again there was evidence that the C-α_1_ splice variant and the canonical α_1_-subunit heterodimerize equally well in intact cells.

The results are expressed as means ± SEM of at least three independent experiments. For minimum 20 cells were analyzed each. All results were controlled for their statistical significance by Student's t-test. A value of p<0.01 was considered to be statistically significant.

The magnitude of the change of the fluorescence-lifetime (≈0.3 ns) was expected to prove an interaction.

### Subcellular distribution of C-α_1_

**Figure 9:**
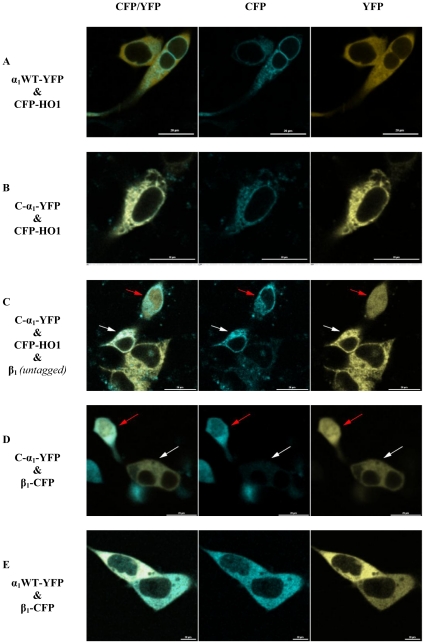
Expression of EYFP-marked α_1_ full-length (A) and C-α_1_ (B) in HEK-293 cells. As a marker of the endoplasmic reticulum [11] ECFP tagged heme oxygenase 1 (human, HO-1) was co-transfected. A, α_1_ full-length shows cytosolic distribution. B, C-α_1_ shows a similar distribution like ECFP-HO-1. C, addition of an untagged β_1_-subunit led in some cells a similar subcellular distribution as the wild type. The red arrow denotes a cell likely co-transfected with β_1_, the white arrow denotes a cell likely not co-transfected with β_1_. D, only cells which express both subunits show homogenous subcellular distribution. The red arrow denotes a cell co-transfected with β_1_, the white arrow denotes a cell not co-transfected with β_1_. E, The subcellular localization of α_1_ full-length is not affected through coexpression of β_1_. Bar, 20 µm. CFP, ECFP channel; YFP, EYFP channel.

While performing expression controls for the FLIM-FRET experiments, we noticed a different subcellular localization of the fluorescent fusion proteins of the C-α_1_ splice variant versus the canonical α_1_ subunit each expressed in the absence of the β_1_-subunit. Because of the granular perinuclear appearance of the splice variant, we co-expressed human heme oxygenase 1 (HO-1) with an amino-terminal ECFP as a well-known marker for the endoplasmic reticulum [11] (Fig. 9A and B). While the C-α_1_ splice variant showed an exact co-localization with HO-1, the canonical α_1_ subunit showed a diverging homogenous distribution in the cytosol (see Fig. 9A and B). Co-expression of untagged β_1_-subunit led to a homogenous wild type like distribution of the C-α_1_ splice variant with an additional nuclear signal in some (Fig. 9C, red arrow) but not all cells (Fig. 9C, white arrow). Co-expression of CFP-tagged β_1_-subunit (and omission of CFP-HO1) demonstrates that expression of substantial amounts of the β_1_-subunit induces this translocation to the nucleus (Fig. 9D, red arrow). In contrast, no nuclear expression was detected for the canonical α_1_ subunit in the presence of β_1_ (Fig. 9E).

**Figure 10:**
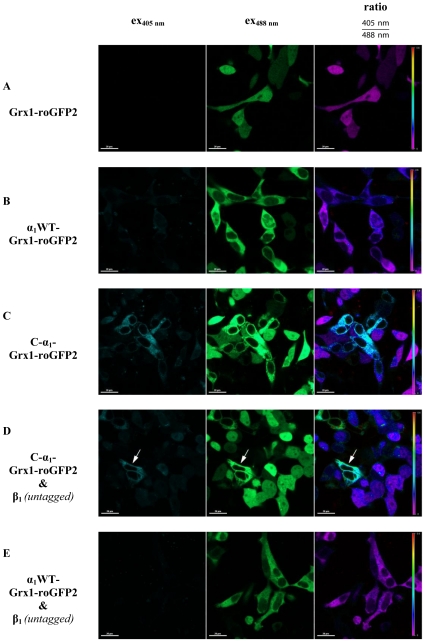
Redox-state analysis of the α_1_ full-length and C-α_1_ in HEK-293 cells, using the redox-sensor Grx1-roGFP2. According to the settings a higher value means a more oxidized state of the protein.

Because *Sharin* et al. [5] have shown that the C-α_1_ splice variant is “uniquely resistant to oxidative protein degradation” we analyzed the redox-state of both α_1_ variants by fusion with the redox-sensor Grx1-roGFP2. Ratiometric analysis showed no difference between Grx1-roGFP2 (Fig. 10A) and α_1_-Grx1-roGFP2 (Fig. 10B). In contrast, C-α_1_-Grx1-roGFP2 showed a higher ratiometric signal indicative of a more oxidized subcellular environment (Fig. 10C). This effect was attenuated by co-expression of the untagged β_1_-subunit (Fig. 10D). In contrast, co-expression of untagged β_1_-subunit with α_1_-Grx1-roGFP2 led to no change (Fig. 10E in comparison to Fig. 10B).

## Discussion

It has recently been shown that the α_1_-subunit of the nitric oxide receptor NOsGC undergoes splicing regulation in differentiating human embryonic cells [5]. The results indicated high levels of an amino-terminally deleted C-α_1_-splice form in differentiating cells that showed a different intracellular distribution in comparison to the canonical full-length α_1_-subunit. It has been published that the amino-terminus of the α_1_-subunit is important for quantitative dimerization with the β_1_-subunit [2]. This was in contrast to a study where we had shown co-purification of the β_1_-subunit with α_1_ΔN_259_ [3]. To explain the discrepancy, *Wagner* et al. [2] raised the argument that we had applied a purification method, where we pooled the fractions with catalytic activity [3]. Due to this approach, it was conceivable, that only a small fraction which has formed active heterodimers is considered, while non-heterodimerizing subunits are discarded. The present study circumvents this potential problem by using a purification procedure based on an affinity StrepTag attached to the β_1_-subunit in analogy to the study of *Wagner* et al. [2]. Our results obtained by using co-purification of the α_1_-subunit and the amino-terminally deleted C-α_1_-splice form with the StrepTagged β_1_-subunit indicate that the C-α_1_-splice form heterodimerizes quantitatively. Because of the controversial nature of the question, whether dimerization of nitric oxide sensitive guanylyl cyclase requires the α_1_ amino-terminus, we used an additional method based on fluorescence resonance energy transfer to demonstrate heterodimerization of the C-α_1_-splice form with the β_1_-subunit in intact cells. This corroborated our finding that lack of the amino-terminus of the α_1_-subunit does not influence quantitative dimerization with the β_1_-subunit. This is also consistent with a number of other studies [4], [12], [13], [14]. Our finding that α_1_-variants heterodimerize equally well in intact cells, but the C-α_1_ splice form seems to lead to more intact heterodimers upon purification is probably due to the greater stability of the C-α_1_ splice variant [5]. This may also be the reason for the higher enzyme activity of the C-α_1_/β_1_ heterodimer versus wild type. Alternatively, it is conceivable, that the amino-terminal region absent in C-α_1_ has a negative regulatory influence on basal activity. This would be in line with a proposed regulatory domain-scale mechanism of the amino-terminal domains of NOsGC [15] that are in proximity to the catalytic domain [9]. The splice variant would thus represent a disinhibited isoform under basal conditions.

Using fluorescent fusion proteins, we noticed a peculiar localization of the C-α_1_-splice form compared to the wild type. Comparison with fluorescent tagged heme oxygenase-1 which is known to be attached to the endoplasmic reticulum outer membrane [16], revealed a highly similar subcellular distribution. As the endoplasmic reticulum shows a relatively oxidizing thiol-disulfide milieu [17], we employed a novel method to measure the glutathione redox potential based on a fusion of the α_1_-subunit in its different splice forms with glutaredoxin-1 and roGFP2 [18]. This confirmed a more oxidizing thiol-disulfide milieu for the C-α_1_ splice variant compatible with localization at the endoplasmic reticulum. Co-expression of the non-tagged β_1_-subunit changed the subcellular localization as well as the thiol-disulfide milieu signal for the C-α_1_-splice form but had no effect on the α_1_-subunit. The finding that the α_1_/β_1_-heterodimer showed the expected diffuse cytosolic localization, while the C-α_1_/β_1_-heterodimer was also present in the nucleus, is interesting in the context of reports suggesting a role for the β_1_-subunit in the nucleus: *Baltrons* and colleagues have demonstrated that the β_1_-subunit translocates to the nucleus for proteosomal degradation in rat astrocytes after treatment with bacterial endotoxin [19]. In a subsequent report the β_1_-subunit was shown to be peripherally associated to chromosomes during mitosis and to play a role in chromatin condensation and cell cycle progression in rat C6 glioma cells [20]. The mechanism by which the β_1_-subunit enters the nucleus is unknown since the protein lacks a recognizable nuclear localization signal [19]. However, it is conceivable that regions of the β_1_-subunit that may be shielded in the classical α_1_/β_1_-heterodimeric enzyme are exposed in the C-α_1_/β_1_-heterodimer and the β_1_-homomer and are free to interact with proteins that regulate nuclear import [21].

*Sharin* et al. have analyzed the subcellular distribution of the native C-α_1_ splice form in differentiating human embryonic cells [5]: With biochemical methods the C-α_1_ splice form could be detected in small amounts in the fraction containing nuclei and to a greater extent in the cytosolic fraction [5]. This is consistent with our findings for the overexpressed fluorescent tagged C-α_1_/β_1_-heterodimer. Immunocytochemical analysis of differentiating human embryonic cells at day 12 with an antibody that recognizes both the α_1_-subunit and the C-α_1_ splice form showed a majority (88 %) of cells with a diffuse staining indicative of a predominant localization in the cytosol [5]. Again this is in line with our results for the fluorescent tagged C-α_1_/β_1_-heterodimer and the fluorescent tagged α_1_/β_1_-heterodimer. In a minority of cells (12 %), a filamentous staining was observed in human embryonic cells using an antibody that recognizes both the α_1_-subunit and the C-α_1_ splice form. These cells were not analyzed for the β_1_-subunit expression. So it remains a possibility that these cells do express no or less β_1_-subunit. This would be consistent with our finding that the fluorescently tagged C-α_1_ splice form shows a different subcellular localization and redox environment alone than in the presence of the β_1_-subunit.

The endoplasmic reticulum is involved in heme trafficking, heme degradation and heme insertion into hemoproteins in eukaryotic cells [22]. Biochemical analysis in native human embryonic cells showed small amounts of the canonical α_1_-subunit in the fraction containing the endoplasmic reticulum. The function of the H-NOX domain of the α-subunits that is absent in the C-α_1_ splice variant is not clear. Very recently it has been suggested based on overexpression and purification of the H-NOX-α_1_-fragment in *E. coli* that it may bind heme via a non-covalent interaction [23]. It is possible that the lack of this domain in the C-α_1_ protein leads to trapping of the splice variant at the site of heme insertion, while the canonical α_1_-subunit or the α_1_/β_1_-heterodimer interacts only transiently with the endoplasmic reticulum e.g. during heme insertion or maturation.

*Sharina* et al. showed that the C-α_1_ splice variant is more stable in intact cells in the presence of ODQ, a well-known oxidant [24] while the canonical α_1_-subunit is more rapidly degraded under these conditions. We show in the current paper that fluorescent labeled overexpressed C-α_1_ protein is targeted to a more oxidized environment in comparison to the canonical α_1_-subunit. Thus the higher stability of the C-α_1_ protein in the presence of oxidants may represent an adaption to the redox environment at the endoplasmic reticulum because it is well known to be a more oxidized compartment than the cytosol [6]. NOsGC activators, like cinaciguat, which act at the H-NOX domain of the β_1_-subunit enhance the stability of the protein through inhibition of proteosomal degradation which can be induced by oxidants like ODQ in the cell [25], [26]. It is thus conceivable that heme or ligand free H-NOX domains not only of β_1_ but also of α_1_ subunits provide a signal that leads to more rapid degradation.

In summary, we present evidence that the amino-terminal H-NOX domain of the α_1_-subunit does not preclude quantitative dimerization with the β_1_-subunit to form a stable active, heme containing NOsGC-heterodimer but is important for subcellular localization.

## Materials and Methods

### Materials

Cinaciguat (BAY 58-2667) was a generous gift from *Johannes-Peter Stasch* (Bayer Pharma AG, Wuppertal, Germany). The *Sf*-9 cells were obtained from Invitrogen (Karlsruhe, Germany), the HEK-293 cells were obtained from the German Collection of Microorganisms and Cell Cultures (DSMZ) (Brunswick, Germany) and the *Sf*-9 Easy Titer cell line (*Sf-*9ET) was a generous gift from *Dr. Dominic Esposito* (National Institutes of Health, Rockville, USA). D-desthiobiotin, avidin and *Strep*-Tactin® Superflow® high capacity resin were purchased from IBA, Goettingen, Germany. 2-diethyl-1-nitroso-oxyhydrazine (DEA/NO), 2-(4-Hydroxyphenylazo)benzoic acid (HABA), creatine kinase, hemin, lipid medium supplement and all other chemicals, in the highest grade of purity, were obtained from Sigma-Aldrich, Munich, Germany. [α-^32^P]GTP (400 Ci/mmol) was purchased from Hartmann Analytic, Brunswick, Germany. All primers used for site directed mutagenesis, were obtained in HPLC purity grade from biomers.net, Ulm, Germany.

### Cloning of α_1_ Deletion Mutants

For construction of the α_1_ΔN_236_ mutant, a *Pvu*II/*Hind*III fragment of the α_1_ full-length (see ref. [3]) clone was ligated *Stu*I/*Hind*III into pFastBac™1vector (Invitrogen, Karlsruhe, Germany). Cloning of the human amino-terminal deletion mutant α_1_ΔN_259_ has been described previously [3].

### Cloning of carboxy-terminal Strep Tag II β_1_-subunit (Sf-9 system)

For construction of the carboxy-terminal *Strep* Tag II with the β_1_-subunit a *Pvu*I/*Stu*I fragment of the conjoined NOsGC construct β_1_α_1_-*Strep* [27] was cloned using *Pvu*I/*Sma*I into the pFastBac™1 vector.

### Cloning of α_1_WT and C-α_1_ fused with fluorescent proteins for determination of fluorescence lifetime

For construction of α_1_ full-length in pECFP-N1 (Clontech, Mountain View, CA, USA) the α_1_ cDNA in pcDNA3.1/V5/His-TOPO described by *Haase* et al. [27] was cloned *Hin*dIII/*Xho*I into *Hin*dIII/*Sal*I pECFP-N1. Cloning of the C-α_1_ (rat α_1_ΔN_258_) was done by introduction of suitable restriction sites by site directed mutagenesis into this construct, restriction and religation. At the same time the *Kozak* consensus sequence [28] was optimized and a start-methionine had to be introduced. For construction of C-α_1_-ECFP the following primer pair was used: 5′-GAA CCA GCC CTA TTT GCT CGA GTC GGT CGC C**AT** **G** GA GAG CAC CAA GCC TTC TCT-3′ and 5′-AGA GAA GGC TTG GTG CTC TC **C AT**G GCG ACC GAC TCG AGC AAA TAG GGC TGG TTC-3′. The initiation codons or complementary sequence are bold type and the modified nucleotides are underlined. Using the respective restriction sites in pECFP before the insert *Xho*I digestion and religation led to C-α_1_-ECFP. Due to a slight difference in the amino acid sequence rat α_1_ΔN_258_ correspond to human α_1_ΔN_259_ (Data S1).

### Cloning of β_1_ fused with EYFP for determination of fluorescence lifetime

The cloning has been described previously [27].

### Exchange of ECFP and EYFP

The carboxy-terminal ECFP-fusions of α_1_ full-length and C-α_1_ were exchanged for EYFP and the carboxy-terminal EYFP-fusion of β_1_ was exchanged for ECFP using the pEYFP-N1 or pECFP-N1 vector (Clontech) and *Age*I/*Bsr*GI as restriction enzymes.

### Cloning of β_1_ in pcDNA3.1/V5/His-TOPO for Expression in HEK-293-cells

The cloning has been described previously [27].

### Cloning of ECFP-HO1 in pECFP-C1

The human heme oxygenase 1 (HO1) was amplified using the primer pair (5′-CCC AGC ACC GGC CGG ATG GAG-3′/5′-TTC AGT GCC CAC GGT AAG GAA GC-3′) und the FirstChoice™ PCR-Ready Human Placenta cDNA (Ambion, Austin, USA). The PCR product was subcloned into pCR®2.1-TOPO® vector. Through *Eco*RI/*Xba*I restriction the insertion into pFastBac™1 was performed. Using *Eco*RI/*Kpn*I the cDNA was transferred into pECFP-C1 [29].

### Cloning of Grx1-roGFP2-tagged α_1_ variants

Grx1-roGFP2 in pLPCX (Clontech) was a kind gift from *Dr. Tobias Dick* (DKFZ, Heidelberg, Germany) [18]. Through restriction with *Bgl*II/*Bsr*GI the construct was ligated into pEGFP-N1. Using *Sma*I/*Xba*I ECFP in α_1_-ECFP (s.a.) was exchanged for Grx1-roGFP2 out of pLCPX (*Eco*47III/*Xba*I restricted). After *Apa*I restriction and religation the frame was restored.

Restriction of C-α_1_-EYFP and α_1_-Grx1-roGFP2 with *Eco*47III/*Bst*EII led to an exchange of the full-length construct for the splice variant.

### Control of sequences

All cloned constructs were verified by sequencing (GATC Biotech, Konstanz, Germany).

### Baculovirus Generation

Recombinant baculoviruses of respective subunits were generated using the Bac-to-Bac® Baculovirus Expression System (Invitrogen).

### Sf-9 Cell Culture, Expression of Recombinant Guanylyl Cyclase Subunits and Preparation of Cytosolic Fraction

*Sf-*9 cells were cultured in *Sf*-900™ II serum-free medium (Invitrogen) supplemented with 1 % penicillin/streptomycin (PAA Laboratories, Coelbe, Germany) and 10 % fetal bovine serum (Foetal Bovine Serum Gold, EU-approved, PAA Laboratories). Spinner cultures were grown at 27°C at 140 rpm shaking on a 50 mm orbit platform and diluted to 2 • 10^6^ cells/ml for infection. 500 ml of cell solution were infected with the respective recombinant baculovirus stock with the multiplicities of infection (MOI) of 1. The MOI was determined using *Sf*-9ET cell line according to the recent published paper by *Hopkins* and *Esposito* [30]. After 72 h cells were harvested by centrifugation (4,000 *g* for 1 min at 4°C). All following steps were performed on ice. The cell pellet was resuspended in 30 ml of lysis buffer containing 50 mM TEA-HCl, 1 mM EDTA, 10 mM dithiothreitol (DTT), 250 nM Avidin, pH 7.4, and complete EDTA-free Protease Inhibitor Cocktail Tablets (Roche, Mannheim, Germany). The cells were lysed by sonication. Cytosolic fractions were obtained by centrifugation for 180 min at 15,000 *g* at 4 °C. The cytosolic fractions were filtered through a 0.2 µm syringe filter (Sartorius, Goettingen, Germany). 2 ml aliquots of cytosolic fractions were kept as reference for experiments to monitor the purification at all steps. For investigation of the influence of heme supplement and additionally of lipid medium supplement 4 mg/l hemin or/and 1 % lipid medium supplement were added.

### One-Step-Purification of NOsGC

The purification was performed on ice or at 4°C. The chromatographic step was performed on an ÄKTApurifier 100 system (GE Healthcare, Munich, Germany). Cytosolic fractions were immediately applied to a *Strep*-Tactin® Superflow® high capacity resin (2 ml Volume in a Tricorn™ 10/20 column) at 1 ml/min [31]. Buffer W contained 100 mM Tris-HCl, 1 M NaCl, 1 mM EDTA, 1 mM benzamidine, 10 mM DTT, pH 8.0. Buffer E was prepared by adding 2.5 mM D-desthiobiotin to buffer W. The column was washed ad 1 ml/min with buffer W for 5 column volumes (CV). With 5 CV buffer E the elution of the NOsGC was performed. Monitoring the absorbance at 254 nm, 280 nm and 430 nm showed a single peak in the fraction which contained NOsGC. Regeneration of the column was performed by 15 CV buffer R (100 mM Tris-HCl, 150 mM NaCl, 1 mM EDTA, 1 mM HABA, pH 8.0) at 1 ml/min and 8 CV of buffer W at 1 ml/min The pooled fractions (approximately 15 ml) were concentrated to a volume of about 500 µl using an Amicon Ultra-15 centrifugal filter unit with 30 kDa cut-off (Millipore, Schwalbach, Germany).

### Determination of Protein Concentration and Guanylyl Cyclase Activity Assay

Protein concentrations were determined by the *Warburg*-*Christian* method [32] using a NanoPhotometer™ (Implen, Munich, Germany). Guanylyl cyclase activity was measured as described previously [3]. Purified NOsGC was diluted with 50 mM TEA-HCl, 10 mM DTT, 1 mM EDTA, 0.5 µg/µl bovine serum albumin (Roth, Karlsruhe, Germany), pH 7.4, quick-frozen in liquid nitrogen with 10 % (v/v) glycerol and stored at -80°C. Enzyme activity of purified protein (50 ng of protein per assay tube) were determined by incubation for 10 min at 37°C in the presence of 1 mM cGMP, 0.5 mM [α-^32^P]GTP (about 0.2 µCi), 3 mM MgCl_2_, 50 mM TEA-HCl, pH 7.4, 0.25 g/liter creatine kinase, 5 mM creatine phosphate, and 1 mM 3-isobutyl-1-methylxanthine in a total volume of 100 µl as described by *Schultz* and *Boehme* [33]. Reactions were started by the addition of protein and incubation at 37°C. All experiments were stopped by ZnCO_3_ precipitation, and purification of the enzyme-formed cGMP was performed as described previously. Basal enzyme activity measurements were performed in the absence of NO or cinaciguat. Measurements of stimulated enzyme were performed in the presence of the NO donor DEA/NO or cinaciguat. DEA/NO was dissolved in 10 mM NaOH, which did not affect the enzyme activity (data not shown). Cinaciguat was dissolved in 100 % DMSO and then diluted in distilled water to a final concentration of 10 µM so that the final DMSO concentration in the enzyme assay did not exceed 2.5% (v/v). At this concentration no effects of DMSO on enzyme activity were observed (data not shown).

### SDS-polyacrylamide electrophoresis gels and immunoblot analysis

Aliquots of 50 µg (cytosolic fractions) or 5 µg (purified enzyme) protein were heated for 3 min at 105°C with lidheat of 120°C (PCR-Cycler) in a modified Laemmli sample buffer (50 mM Tris-HCl, 1% SDS, 100 mM DTT, 30% Glycerol, pH 7.5). After the heat-incubation 1 µl of a blue sample puffer [10% (m/w) bromophenol blue solved in the modified Laemmli buffer] were added and the probes were resolved on 10% slab gels. Proteins were stained according to *Kang* et al. 2002 [8]. As protein markers predominantly PageRuler™ Prestained Protein Ladder and PageRuler™ Unstained Protein Ladder from Fermentas (St. Leon-Rot, Germany) were used. For immunoblotting, protein fractions were transferred electrophoretically to a nitrocellulose membrane [Amersham™ Hybond™ ECL (GE Healthcare)]. The membrane was reversibly stained with Ponceau S to evaluate the protein transfer. Unspecific binding sites were saturated by immersing the membrane for 1 h at room temperature in TBST (10 mM Tris-HCl, pH 8.0, 150 mM NaCl, 0.1% (v/v) Tween 20) containing 5% nonfat dry milk. The following antibodies against the two NOsGC subunits were used for detection: anti-α_1_ (1∶5,000) (Sigma, G4280) or α_1_-1200 (1∶1,000; as described in [3]) and anti-β_1_ (1∶4,000) (Sigma, G4530). Antibodies were incubated for 1 h in TBST-buffer at room temperature. The membranes were washed three times for 10 min with TBST and subsequently incubated for 1 h with horseradish-peroxidase-conjugated anti-rabbit IgG antibody (1∶2,000/Cell Signaling Technology, distributor: New England Biolabs, Frankfurt am Main, Germany or 1∶4,000/Sigma). After three washes with TBST-buffer the membranes were processed with the enhanced chemiluminescence western blotting detection system according to the recommendations of the manufacturer (Roche) and the signals were detected with a charge-coupled device camera (Intas, Goettingen, Germany) or the membranes were processed with ECL Western blotting detection system according to manufacturer's recommendations (Amersham Pharmacia Biotech, Piscataway, USA).

### Quantification of heterodimerization

For quantification of subunit interaction, the Coomassie stained gels were either captured using a white trans-illuminator and a charge-coupled device camera (HighRes, Intas) or scanned using ScanMaker i900 (Microtek, Willich, Germany). The analysis was done using LabImage 1D (Kapelan, Leipzig, Germany). For the wild-type of the human α_1_-subunit and C-α_1_ the densitometric values of α_1_ full-length and the truncation were normalized to the value of β_1_ in the same lane and dimerization is given as a percentage of α_1_WT dimerization, which was set to 100%. Western blots were analyzed accordingly with similar results (data not shown).

### Fluorescence Lifetime Imaging (FLIM) using a confocal laser scanning microscope

Determination of fluorescence lifetime was done as described previously [9]. For microscopy HEK-293 cells were seeded in 24-well imaging plates with special glass bottom (zell-kontakt, Noerten-Hardenberg, Germany; distributed by PAA Laboratories, Coelbe, Germany) and transfected with the cDNA coding for the respective constructs for the expression of fluorescent tagged NOsGC subunits using Lipofectamin™ LTX (Invitrogen). 48 h post transfection cells were imaged at 37°C on a Nikon Ti-E microscope equipped with an incubation chamber (Okolab, Naples, Italy) using a 60-fold immersion objective (NA 1.4, Nikon). For live cell imaging culture medium was removed, cells were washed twice and supplemented with Hank's balanced salt solution.

Fluorescence decays were measured in cells expressing the FRET donor alone (ECFP) using the vector pECFP-N1 (Clontech) as a negative control. The rat α_1_-subunits, wild-type and amino-terminal truncation are carboxy-terminally tagged with ECFP as FRET donor and the rat β_1_-subunit is carboxy-terminally tagged with FRET acceptor EYFP using the vector pEYFP-N1 (Clontech) or vice versa. Images were collected using a 405 nm pulsed laser. Emitted fluorescence signals were selected using a CFP bandpass filter (475/20 nm). FLIM images in the time domain from fluorescent cells were recorded with a 4 channel time gated detection system (LiMo module, Nikon). The images were 256×256 pixels in size and the acquisition time was 5 min.

### Localization of α_1_ constructs by confocal laser scanning microscope

The cells were prepared as described above. Amino-terminally ECFP-tagged heme oxygenase 1 (human, HO-1) was used as a marker for the endoplasmic reticulum [11].

### Analyzing the redox state of the C-α_1_ and the α_1_ wild type protein

Using fusion proteins of the respective α_1_ isoforms with the redox sensor Grx1-roGFP2 we performed a ratiometric measurement. The cells were simultaneously excited with a 405 nm and a 488 nm laser. The emission was collected by a GFP bandpass filter (525/50 nm). The ratio of the 405/488 emission corresponds to the redox state. A high value means a more oxidized state and vice versa.

### Statistical analysis

The results are expressed as means ± SEM of at least three independent experiments. All results were controlled for their statistical significance by Student's *t*-test. A value of *p*<0.01 was considered to be statistically significant.

## Supporting Information

Data S1 **Amino acid sequence alignment of human (Accession number: NP_000847.2) and rat (Accession number: NP_058786.2) α_1_-subunit to show sequence differences (performed with ClustalW2 at** www.ebi.ac.uk **).** Gaps are marked.
(PDF)

Data S2 Nucleic acid sequence alignment (partly shown) of human α_1_ full-length (GB: Y15723) and both splice-variants C*-α_1_ (GB: BX649180)/C-α_1_ (GB: AK226125) (performed with ClustalW2 at www.ebi.ac.uk **).** Initiation codons are marked.
(PDF)
