# Supplementary material for: The Amino-Terminus of Nitric Oxide Sensitive Guanylyl Cyclase α1 Does Not Affect Dimerization but Influences Subcellular Localization
Source: PLoS One. 2011 Sep 30;6(9):e25772. doi: 10.1371/journal.pone.0025772 (PMC3184163; doi:10.1371/journal.pone.0025772)
Supplement: Data S1 — Amino acid sequence alignment of human (Accession number: NP_000847.2) and rat (Accession number: NP_058786.2) α1-subunit to show sequence differences (performed with ClustalW2 at www.ebi.ac.uk ). Gaps are marked. (PDF) [file pone.0025772.s001.pdf]

S1

```
α1 (human) (1)    MFCTKLKDLKITGECPFSLLAPGQVPNESSEEAAGSSESCKATVPICQDI
α1 (rat)   (1)    MFCRKFKDLKITGECPFSLLAPGQVPTEPIEEVAGVSESCQATLPTCQEF

α1 (human) (51)   PEKNIQESLPQRKTSRSRVYLHTLAESICKLIFPEFERLNVALQRTLAKH
α1 (rat)   (51)   AEN-AEGSHPQRKTSRNRVYLHTLAESIGKLIFPEFERLNLALQRTLAKH

α1 (human) (101)  KIKESRKSLEREDFEKTIAEQAVAAGVPVEVIKESLGEEVFKICYEEDEN
α1 (rat)   (100)  KIKENRNSSEKEDLERIIAEEAIAAGVPVEALKDSLGEELFKICYEEDEH

α1 (human) (151)  ILGVVGGTLKDFLNSFSTLLKQSSHCQEAGKRGRLEDASILCLDKEDDFL
α1 (rat)   (150)  ILGVVGGTLKDFLNSFSTLLKQSSHCQEAERRGRLEDASILCLDKDQDFL

α1 (human) (201)  HVYYFFPKRTTSLILPGIIKAAAHVLYETEVEVSLMPPCFHNDCSEFVNQ
α1 (rat)   (200)  NVYYFFPKRTTALLLPGIIKAAARILYESHVEVSLMPPCFRSECTEFVNQ

α1 (human) (251)  PYLLYSVHMKSTKPSLSPSKPQSSLVIPTSLFCKTFPFHFMFDKDMTILQ
α1 (rat    (250)  PYLLYSVHVKSTKPSLSPGKPQSSLVIPTSLFCKTFPFHFMLDRDLAILQ

α1 (human) (301)  FGNGIRRLMNRRDFQGKPNFEEYFEILTPKINQTFSGIMTMLNMQFVVRV
α1 (rat)   (300)  LGNGIRRLVNKRDFQGKPNFEEFFEILTPKINQTFSGIMTMLNMQFVIRV

α1 (human) (351)  RRWDNSVKKSSRVMDLKGQMIYIVESSAILFLGSPCVDRLEDFTGRGLYL
α1 (rat)   (350)  RRWDNLVKKSSRVMDLKGQMIYIVESSAILFLGSPCVDRLEDFTGRGLYL

α1 (human) (401)  SDIPIHNALRDVVLIGEQARAQDGLKKRLGKLKATLEQAHQALEEEKKKT
α1 (rat)   (400)  SDIPIHNALRDVVLIGEQARAQDGLKKRLGKLKATLEHAHQALEEEKKKT

α1 (human) (451)  VDLLCSIFPCEVAQQLWQGQVVQAKKFSNVTMLFSDIVGFTAICSQCSPL
α1 (rat)   (450)  VDLLCSIFPSEVAQQLWQGQIVQAKKFNEVTMLFSDIVGFTAICSQCSPL

α1 (human) (501)  QVITMLNALYTRFDQQCGELDVYKVETIGDAYCVAGGLHKESDTHAVQIA
α1 (rat)   (500)  QVITMLNALYTRFDQQCGELDVYKVETIGDAYCVAGGLHRESDTHAVQIA

α1 (human) (551)  LMALKMMELSDEVMSPHGEPIKMRIGLHSGSVFAGVVGVKMPRYCLFGNN
α1 (rat)   (550)  LMALKMMELSNEVMSPHGEPIKMRIGLHSGSVFAGVVGVKMPRYCLFGNN

α1 (human) (601)  VTLANKFESCSVPRKINVSPTTYRLLKDCPGFVFTPRSREELPPNFPSEI
α1 (rat)   (600)  VTLANKFESCSVPRKINVSPTTYRLLKDCPGFVFTPRSREELPPNFPSDI

α1 (human) (651)  PGICHFLDAYQQ-GTNSKPCFQKKDVEDGNANFLGKASGID
α1 (rat)   (650)  PGICHFLDAYQHQGPNSKPWFQQKDAEDGNANFLGKASGVD
```
