# Supplementary material for: The Amino-Terminus of Nitric Oxide Sensitive Guanylyl Cyclase α1 Does Not Affect Dimerization but Influences Subcellular Localization
Source: PLoS One. 2011 Sep 30;6(9):e25772. doi: 10.1371/journal.pone.0025772 (PMC3184163; doi:10.1371/journal.pone.0025772)
Supplement: Data S2 — Nucleic acid sequence alignment (partly shown) of human α1 full-length (GB: Y15723) and both splice-variants C*-α1 (GB: BX649180)/C-α1 (GB: AK226125) (performed with ClustalW2 at www.ebi.ac.uk ). Initiation codons are marked. (PDF) [file pone.0025772.s002.pdf]

S2

```
                                                 ATG_1
α1-WT    (496)  TCGCAGCAGGGTAAGAGACACCAACACCATGTTCTGCACGAAGCTCAAGG
C*-α1    (276)  TCGCAGCAGGGTAAGAGACACCAACACCATGTTCTGCACGAAGCTCAAGG
C-α1     (167)  --------------------------------------------------

α1-WT    (546)  ATCTCAAGATCACAGGAGAGTGTCCTTTCTCCTTACTGGCACCAGGTCAA
C*-α1    (326)  ATCTCAAGATCACAGGAGAGTGTCCTTTCTCCTTACTGGCACCAGGTCAA
C-α1     (167)  ---------------------------------------------GTCAA

α1-WT    (596)  GTTCCTAACGAGTCTTCAGAGGAGGCAGCAGGAAGCTCAGAGAGCTGCAA
C*-α1    (376)  GTTCCTAACGAGTCTTCAGAGGAGGCAGCAGGAAGCTCAGAGAGCTGCAA
C-α1     (172)  GTTCCTAACGAGTCTTCAGAGGAGGCAGCAGGAAGCTCAGAGAGCTGCAA

α1-WT    (646)  AGCAACCGTGCCCATCTGTCAAGACATTCCTGAGAAGAACATACAAGAAA
C*-α1    (426)  AGCAACCGTGCCCATCTGTCAAGACATTCCTGAGAAGAACATACAAGAAA
C-α1     (222)  AGCAACCGTGCCCATCTGTCAAGACATTCCTGAGAAGAACATACAAGAAA

α1-WT    (696)  GTCTTCCTCAAAGAAAAACCAGTCGGAGCCGAGTCTATCTTCACACTTTG
C*-α1    (476)  GTCTTCCTCAAAGAAAAACCAGTCGGAGCCGAGTCTATCTTCACACTTTG
C-α1     (272)  GTCTTCCTCAAAGAAAAACCAGTCGGAGCCGAGTCTATCTTCACACTTTG

α1-WT    (746)  GCAGAGAGTATTTGCAAACTGATTTTCCCAGAGTTTGAACGGCTGAATGT
C*-α1    (526)  GCAGAGAGTATTTGCAAACTGATTTTCCTAGAGTTTGAACGGCTGAATGT
C-α1     (322)  GCAGAGAGTATTTGCAAACTGATTTTCCCAGAGTTTGAACGGCTGAATGT

α1-WT    (796)  TGCACTTCAGAGAACATTGGCAAAGCACAAAATAAAAGAAAGCAGGAAAT
C*-α1    (576)  TGCACTTCAGAGAACATTGGCAAAGCACAAAATAAAAGAAAGCAGGAAAT
C-α1     (372)  TGCACTTCAGAGAACATTGGCAAAGCACAAAATAAAAGAAAGCAGGAAAT

α1-WT    (846)  CTTTGGAAAGAGAAGACTTTGAAAAAACAATTGCAGAGCAAGCAGTTGCA
C*-α1    (626)  CTTTGGAAAGAGAAGACTTTGAAAAAACAATTGCAGAGCAAGCAGTTGCA
C-α1     (422)  CTTTGGAAAGAGAAGACTTTGAAAAAACAATTGCAGAGCAAGCAGTTGCA

α1-WT    (896)  GCAGGAGTTCCAGTGGAGGTTATCAAAGAATCTCTTGGTGAAGAGGTTTT
C*-α1    (676)  GCAG----------------------------------------------
C-α1     (472)  GCAGGAGTTCCAGTGGAGGTTATCAAAGAATCTCTTGGTGAAGAGGTTTT

α1-WT    (946)  TAAAATATGTTACGAGGAAGATGAAAACATCCTTGGGGTGGTTGGAGGCA
C*-α1    (680)  --------------------------------------------------
C-α1     (522)  TAAAATATGTTACGAGGAAGATGAAAACATCCTTGGGGTGGTTGGAGGCA

α1-WT    (996)  CCCTTAAAGATTTTTTAAACAGCTTCAGTACCCTTCTGAAACAGAGCAGC
C*-α1    (680)  ------------------------------------------AG--CAGC
C-α1     (572)  CCCTTAAAGATTTTTTAAACAGCTTCAGTACCCTTCTGAAACAGAGCAGC

α1-WT   (1046)  CATTGCCAAGAAGCAGGAAAAAGGGGCAGGCTTGAGGACGCCTCCATTCT
C*-α1    (686)  CATTGCCAAGAAGCAGGAAAAAGGGGCAGGCTTGAGGACGCCTCCATTCT
C-α1     (622)  CATTGCCAAGAAGCAGGAAAAAGGGGCAGGCTTGAGGACGCCTCCATTCT

α1-WT   (1096)  ATGCCTGGATAAGGAGGATGATTTTCTACATGTTTACTACTTCTTCCCTA
C*-α1    (736)  ATGCCTGGATAAGGAGGATGATTTTCTACATGTTTACTACTTCTTCCCTA
C-α1     (672)  ATGCCTGGATAAGGAGGATGATTTTCTACATGTTTACTACTTCTTCCCTA

α1-WT   (1146)  AGAGAACCACCTCCCTGATTCTTCCCGGCATCATAAAGGCAGCTGCTCAC
C*-α1    (786)  AGAGAACCACCTCCCTGATTCTTCCCGGCATCATAAAGGCAGCTGCTCAC
C-α1     (722)  AGAGAACCACCTCCCTGATTCTTCCCGGCATCATAAAGGCAGCTGCTCAC

α1-WT   (1196)  GTATTATATGAAACGGAAGTGGAAGTGTCGTTAATGCCTCCCTGCTTCCA
C*-α1    (836)  GTATTATATGAAACGGAAGTGGAAGTGTCGTTAATGCCTCCCTGCTTCCA
C-α1     (772)  GTATTATATGAAACGGAAGTGGAAGTGTCGTTAATGCCTCCCTGCTTCCA
                                                ATG_236
α1-WT   (1246)  TAATGATTGCAGCGAGTTTGTGAATCAGCCCTACTTGTTGTACTCCGTTC
C*-α1    (886)  TAATGATTGCAGCGAGTTTGTGAATCAGCCCTACTTGTTGTACTCCGTTC
C-α1     (822)  TAATGATTGCAGCGAGTTTGTGAATCAGCCCTACTTGTTGTACTCCGTTC

α1-WT   (1296)  ACATGAAAAGCACCAAGCCATCCCTGTCCCCCAGCAAACCCCAGTCCTCG
C*-α1    (936)  ACATGAAAAGCACCAAGCCATCCCTGTCCCCCAGCAAACCCCAGTCCTCG
C-α1     (872)  ACATGAAAAGCACCAAGCCATCCCTGTCCCCCAGCAAACCCCAGTCCTCG
               ATG_259
```
